# Early transcriptomic response of the mycoparasite *Sphaerodes mycoparasitica* to the mycotoxigenic *Fusarium graminearum* 3-ADON, the cause of Fusarium head blight

**DOI:** 10.1186/s40643-021-00479-y

**Published:** 2021-12-16

**Authors:** Seon Hwa Kim, Vladimir Vujanovic

**Affiliations:** Department of Food and Bioproduct Sciences, University of Saskatchewan, 51 Campus Drive, Saskatoon, SK S7N 5A8 Canada

**Keywords:** Functional transcriptomics, Biotrophic mycoparasitism, *Fusarium* biocontrol, Hyphal cell–cell interaction, RNA-Seq, qPCR, Gene expression

## Abstract

Mycoparasites are an assemblage of biotrophic and necrotrophic fungi that occur on plant pathogenic fungal hosts. Biotrophic mycoparasites are often overlooked in transcriptomic-based biocontrol studies. *Sphaerodes mycoparasitica* (*S.m.*) is a specific biotrophic mycoparasite of plant pathogenic *Fusarium graminearum (F.g.)*, a devastating Fusarium head blight (FHB) disease in small-grain cereals. To understand the biotrophic mycoparasitism comprehensively, we performed Illumina RNA-Seq transcriptomic study on the fungus–fungus interaction in vitro. The aim is to identify the transcript-level mechanism related to the biotrophic *S.m.* mycoparasitism, particularly its ability to effectively control the *F.g.* 3-ADON chemotype. A shift in the transcriptomic profile of the mycoparasite was triggered in response to its interaction with *F.g.* during recognition (1.5 days) and colonization (3.5 days) steps. RNA-Seq analysis revealed ~ 30% of annotated transcripts with "function unknown". Further, 14 differentially expressed genes functionally linked to the biotrophic mycoparasitism were validated by quantitative real-time PCR (qPCR). The gene expression patterns of the filamentous haemagglutinin/adhesin/attachment factor as well as cell wall-degrading glucanases and chitinases were upregulated by host interaction. Besides, mycoparasitism-associated antioxidant resistance genes encoding ATP-binding cassette (ABC) transporter(s) and glutathione synthetase(s) were upregulated. However, the thioredoxin reductase was downregulated which infers that this antioxidant gene can be used as a resistance marker to assess *S.m.* antifungal and antimycotoxigenic activities. The interactive transcriptome of *S. mycoparasitica* provides new insights into specific mycoparasitism and will contribute to future research in controlling FHB.

**Graphic Abstract:**

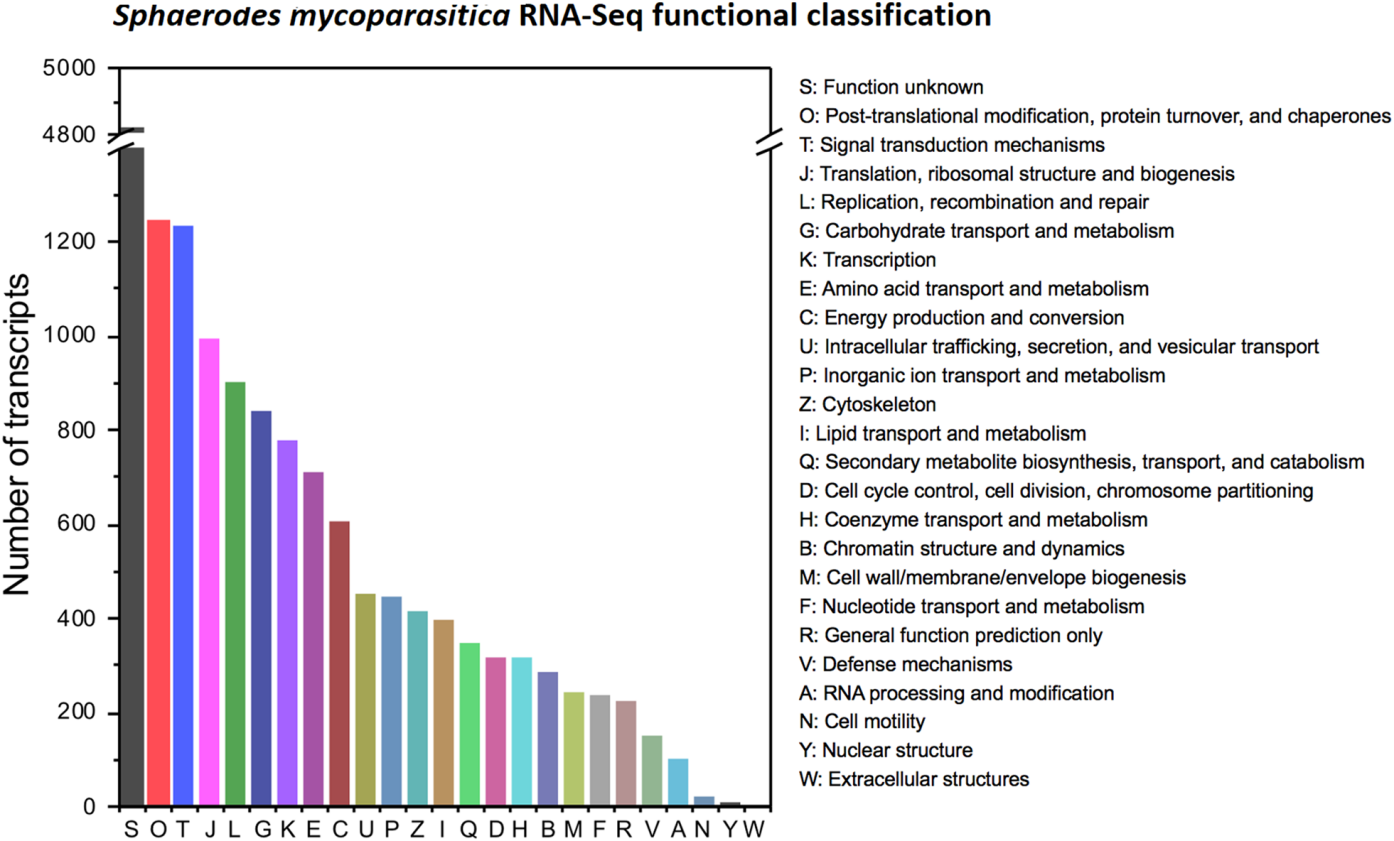

## Introduction

Advances in DNA/RNA-sequencing technologies have provided an opportunity to accelerate research on the beneficial microbiome and pathobiota of plants. *Fusarium graminearum* [teleomorph, *Gibberella zeae* (Schwabe) Petch] (*F.g.*) is the principal causal agent of Fusarium head blight (FHB or scab) disease, resulting in severe losses of small cereal crop yields worldwide (Karlsson et al. 2021; Powell and Vujanovic 2021). *F. graminearum* actively produces various secondary metabolites, and *F.g.* 3-ADON is a particularly virulent chemotype producing abundant mycotoxins, such as zearalenone (ZEN), deoxynivalenol (DON/vomitoxin), its acetyl-derivatives, and aurofusarin (AUR) (Spanic et al. 2020; Birr et al 2021). Despite the economic importance of *F.g.* 3-ADON, effective FHB control and DON detoxification methods are yet to be discovered.

Recent discoveries of fungal antagonists, including mycoparasitic fungi and mycoparasitism-related genes, have provided a basis for controlling *Fusarium* and improving phytoprotection (Moreno-Ruiz 2021; Kim et al. 2021). Indeed, emerging new biocontrol approaches are expected to control diseases and improve breeding programs to increase crop yields and generate more microbe-optimized crops (Trivedi et al. 2017; Vujanovic 2021). Biocontrol is seen as a promising tool to reduce the use of synthetic chemicals in staple crops while controlling FHB (Legrand et al. 2017). Hence, the integrative research based on the mycoparasite-driven control of FHB and associated toxins could generate new insights. In particular, the suppression of the early growth and reproduction of *F.g.* 3-ADON is a key focus.

The development of highly reliable transcriptomic technologies (Ziegenhain et al. 2017) using cell and tissue-based in vitro assays has opened a new chapter of research on biocontrol agent–phytopathogen interactomes (Nygren et al. 2018). Clarifying the molecular principles of mycoparasite–*Fusarium* interactions at the gene expression level may provide insights into the mechanisms underlying mycoparasitism. *Sphaerodes mycoparasitica* (*S.m.*) SMCD 2220–01, a specific ascomycetous mycoparasite on *Fusaria* (Vujanovic and Goh 2009), shows a diphasic mycoparasitic lifestyle and an ability to rapidly adapt during the mitosporic stage (Vujanovic and Kim 2018) to efficiently control the pathogenic and mycotoxin-producing *Fusarium* hosts (Kim and Vujanovic 2016; Vujanovic and Goh 2010). In addition to the biocontrol effect, this mycopathogen effectively reduces AUR mycotoxin production in red-pigmented *Fusaria* by down-regulating *AUR* gene expression (Vujanovic et al. 2017; Vujanovic and Goh 2011). Although the mycoparasite degrades DON, 3-ADON, 15-ADON, and ZEN (Kim and Vujanovic 2017) and inhibits their production in *F. graminearum* 3-ADON (Vujanovic and Chau 2012; Vujanovic et al. 2012), the mechanism underlying mycoparasitism at the transcriptomic level is still unknown (Vujanovic 2021). In addition to transcripts associated with host recognition, attachment, and infection, several other differentially expressed transcripts during mycoparasitism are still unknown, suggesting that further research is needed to understand the biological processes contributing to effective biocontrol (Kim et al. 2021; Zhao et al. 2020). An important gap of transcriptomic knowledge exists about specific-biotrophic mycoparasitism, while the expression trends of some loci may greatly differ between *S. mycoparasitica* and other mycoparasitic taxa (Alfiky and Weisskopf 2021; Gupta et al. 2014). Hence, an improved understanding of the mycoparasitism of *S. mycoparasitica* on *F. graminearum* could be the basis for the development of more efficient *Fusarium* biocontrol methods.

Recently, Kim and Vujanovic (2018) discovered important changes in mycoparasite–*Fusarium* host interfaces, as revealed by water contact angle and atomic force microscopy (Kim and Vujanovic 2018). In this study, we hypothesized that shifts in functional transcripts exist on the exogenous (interspecific) host interface between *S.m.* and *F.g.* 3-ADON. To date, a reliable RNA-seq library has not been established to evaluate the biotrophic mycoparasitism. Hence, the aim of this research was to relate the biotrophic mycoparasitic behavior of *S.m.*, particularly its ability to effectively control *F.g.* 3-ADON, to its capacity to regulate gene expression during the early recognition (1.5 d) and colonization (3.5 days) events in the process of specific mycoparasitism. The interface-interaction was evaluated by NGS-RNA sequencing technology, with superior performance and sensitivity to discover variation in modified interactive transcripts (Schwartz and Motorin 2017; Tsang et al 2021). To confirm the expression profile obtained by RNA-Seq, 14 functional and randomly selected genes were validated in the passive and active mycoparasite response to the *F.g.* 3-ADON host defense by qRT-PCR.

## Methods

### Fungal growth and treatment

The specific mycoparasite *Sphaerodes mycoparasitica* Vujan. (SMCD 2220–01) and the phytopathogenic and mycotoxigenic host *Fusarium graminearum* Schwabe 3-ADON (SMCD 2243) were used. For general transcriptomic profiling of the mycoparasite during biotrophic mycoparasitism or to induce mycoparasitism-related gene expression, the mycoparasite was co-cultured on top of the host with a monofilament fabric, nylon mesh with 30 µm openings (SEFAR NITEX 03–48/31; Sefar Inc., Depew, NY, USA) placed between the mycoparasite and the host. The used growth medium was potato glucose agar (PGA; Sigma-Aldrich, St. Louis, MO, USA) and co-cultures were incubated at 23 °C. The mycelia of the interactive mycoparasite were collected at both incubation time (1.5 and 3.5 days) used for RNA extraction. The method used in this study allowed to obtain a mycelium of the mycoparasite from co-cultures during an active mycoparasite–host interaction. The control samples consist of mycoparasite’s hyphae grown on the fabric tissue without the host.

### RNA extraction, cDNA library construction and Illumina sequencing

Four different mycelia samples with six biological replicates were subjected to RNA extraction using the Aurum™ Total RNA Mini Kit (Bio-Rad, Hercules, CA, USA) according to the manufacturer’s instructions. The quality and quantity of the extracted total RNA was checked on 1% agarose gels followed by the NanoDrop 2000c spectrophotometer (Thermo Scientific, Wilmington, DE, USA) resulting in all samples having a 260/280 nm ratio of 1.9–2.1 and a 260/230 nm ratio of 2.0–2.3. To validate RNA quality, the RNA integrity number (RIN) was measured using the Bioanalyzer 2100 (Agilent Technologies, Santa Clara, CA, USA). All samples showing RIN values above 8 were used for further poly(A) + RNA purification and cDNA library synthesis using the TrueSeq Stranded mRNA Sample Prep Kit (Illumina, San Diego, CA, USA), conducted at the McGill University and Génome Québec Innovation Center (Montréal, Québec). The libraries were multiplexed with Illumina barcodes and each lane was sequenced using the Illumina HiSeq 4000 (100 bp PE reads) based on the massively parallel sequencing protocol (Xiao et al. 2013).

### De novo transcriptome assembly

Transcriptome de novo assembly from RNA-Seq data was performed at the Canadian Centre for Computational Genomics based on a general pipeline described previously (Grabherr et al. 2011; Haas et al. 2013) using the Trinity assembly software suite (http://trinotate.github.io). Raw reads were trimmed from the 3′ end with a Phred score threshold of 30. Illumina sequencing adapters were removed, and all reads were required to have a length of at least 50 bp. Trimmomatic tool was used for quality trimming and adapter clipping (Bolger et al. 2014). The clean reads were utilized for de novo transcriptome assembly using the Trinity assembler (Grabherr et al. 2011) consisting of three modules: Inchworm, Chrysalis, and Butterfly. Then, Bowtie2 and eXpress generated the length normalized counts (transcripts per million, TPM).

### Functional annotation of transcriptome

BlastX alignment and annotation with an *E*-value cut-off set to 10^–5^ were performed against the National Center for Biotechnology and Information (NCBI) non-redundant protein database (nr), UniProtKB/Swiss-Prot (SwissProt) database (https://www.expasy.org/resources/uniprotkb-swiss-prot), and Uniref90 (https://www.uniprot.org/uniref/) (Apweiler et al. 2004). Further functional annotation was achieved using the Trinotate pipeline (http://trinotate.github.io), including Hmmer v.3.1b1 (http://hmmer.org) and PFAM (Finn et al. 2015) for protein domain identification, SignalP v.4.1 (http://www.cbs.dtu.dk/services/SignalP/) (Petersen et al. 2011). The Tmhmm v.2.0c (http://www.cbs.dtu.dk/services/TMHMM/) was employed for protein signal peptide and transmembrane domain prediction, while the EMBL UniProt eggNOG/GO pathway databases (http://eggnog.embl.de) was used to perform the functional enrichment analyses (Powell et al. 2011).

### Quantitative real-time PCR for validation of specific genes

The expression levels of *S.m.* transcripts that were differentially expressed at different incubation times with and without host interactions were calculated by TPM. A TPM ratio > 2 between the control and mycoparasitism conditions at each incubation time was used to identify gene expression differences. To validate the reliability of the transcriptome of *S.m.*, 14 up- or down-regulated transcripts were randomly selected as mycoparasitism-associated candidates and their expression levels were quantified with and without interactions with the host at each incubation time by quantitative real-time PCR (qPCR), with the internal reference gene 18S rRNA for normalization (Xu et al. 2018). The primer pairs (Table 1) for the genes of interest were designed using Primer3 (Koressaar and Remm 2007; Untergasser et al. 2012). Prior to qPCR, the specificity of the designed primers was checked by conventional PCR with the following program: 94 °C for 2 min; 35 cycles of 94 °C for 30 s, 60 °C for 30 s, and 72 °C for 30 s; followed by 72 °C for 10 min. For qPCR assays, the total RNAs of the four samples were used to synthesize cDNAs using the iScript™ cDNA Synthesis Kit (Bio-Rad, Hercules, CA, USA) and the cDNAs were subjected to the qPCR assays using iTaq Universal SYBR® Green Supermix (Bio-Rad) and the MiniOpticon Real-time PCR Detection System with CFX Manager™ (Bio-Rad) in a 20 μl reaction system following manufacturer’s instructions. Quantitative PCR was performed with three replicates with the following program: 95 °C for 30 s; 40 cycles of 95 °C for 10 s and 60 °C for 30 s. After the reaction, fluorescence values were monitored every 0.5 °C from 65 °C to 95 °C to check for non-specific amplification. The relative expression levels of the transcripts were calculated using the 2^–∆∆Ct^ method (Livak and Schmittgen 2001).

**Table 1:** Primers used in this study

Gene	Predict function	Primers (5′ to 3′)
Sm72993	Glucan 1,3-beta-glucosidase	F: TGTCGGCGAGAACAACTGTC
		R: GTGCCTCCTCAACATCCTACG
Sm77681	Tfo1: an Ac-like transposon	F: AGGGAACCCATTGACTCCTG
		R: AGATACGGACGGCATACAACC
Sm70239	Glutathione synthetase	F: TCCCGCTATCTACTACGCCC
		R: ATTTTGCAGGCAGCGTTAGC
Sm72448	Endochitinase B	F: CTCAGCTAACGCACACAACAG
		R: CATGAGTCGGTGAGATGGCT
Sm76207	Filamentous hemagglutinin/adhesin	F: ACAGGACCAATGTAGCACCC
		R: AGGAGCGTCAGCAAATGAGG
Sm76684	ATP-binding cassette (ABC) transporter	F: CATTTGGGCAGAAACGTGGG
		R: TAATGCAGTGTGCGGGTAGA
Sm78917	Uncharacterized mitochondrial protein ymf40	F: CGCTGGTGGAAATATGTGCG
		R: AAGCTCAGTCCGTTGTAGGC
Sm79801	RNase H domain-containing protein	F: GGCCTAGTGAGCTACGGAAG
		R: TGCCAGGGTGAAAAGAGAGG
Sm80489	Thioredoxin-2	F: ATGCCCACTTTCCTGCTCTA
		R: CCTGGGAAGCCTTGTGTTTTTC
Sm76483	Glutathione S-transferase omega-like 2	F: CGACGTGGGGGTGTATAGTG
		R: CGCAGGATCTCAGACGACTC
Sm79379	Thioredoxin reductase	F: GGGTGAAGAAGGGTTGGAGG
		R: AGCGCCTTTGGCAATTTCTG
Sm73177	NADPH–cytochrome P450 reductase	F: ACGGGATTGGAGGTGGTCTA
		R: CACTACACCGAGATGCGGAA
Sm79054	Uncharacterized protein	F: GCCGCGTGGATTAGAAAAGG
		R: ATAGGCGACAGATGACACGG
Sm75119	Uncharacterized membrane protein YJL163C	F: GGATACTTCCTCGCCACCAG
		R: TGTGCTGAAAACAATGGGCG
18S rRNA	Housekeeping gene as an internal reference	F: CTTCGGGGCTCTCTGGTGAT
	(Xu et al. 2018)	R: TGCTGCCTTCCTTGGATGTAGT

## Results

### COG functional classification

**Fig. 1:**
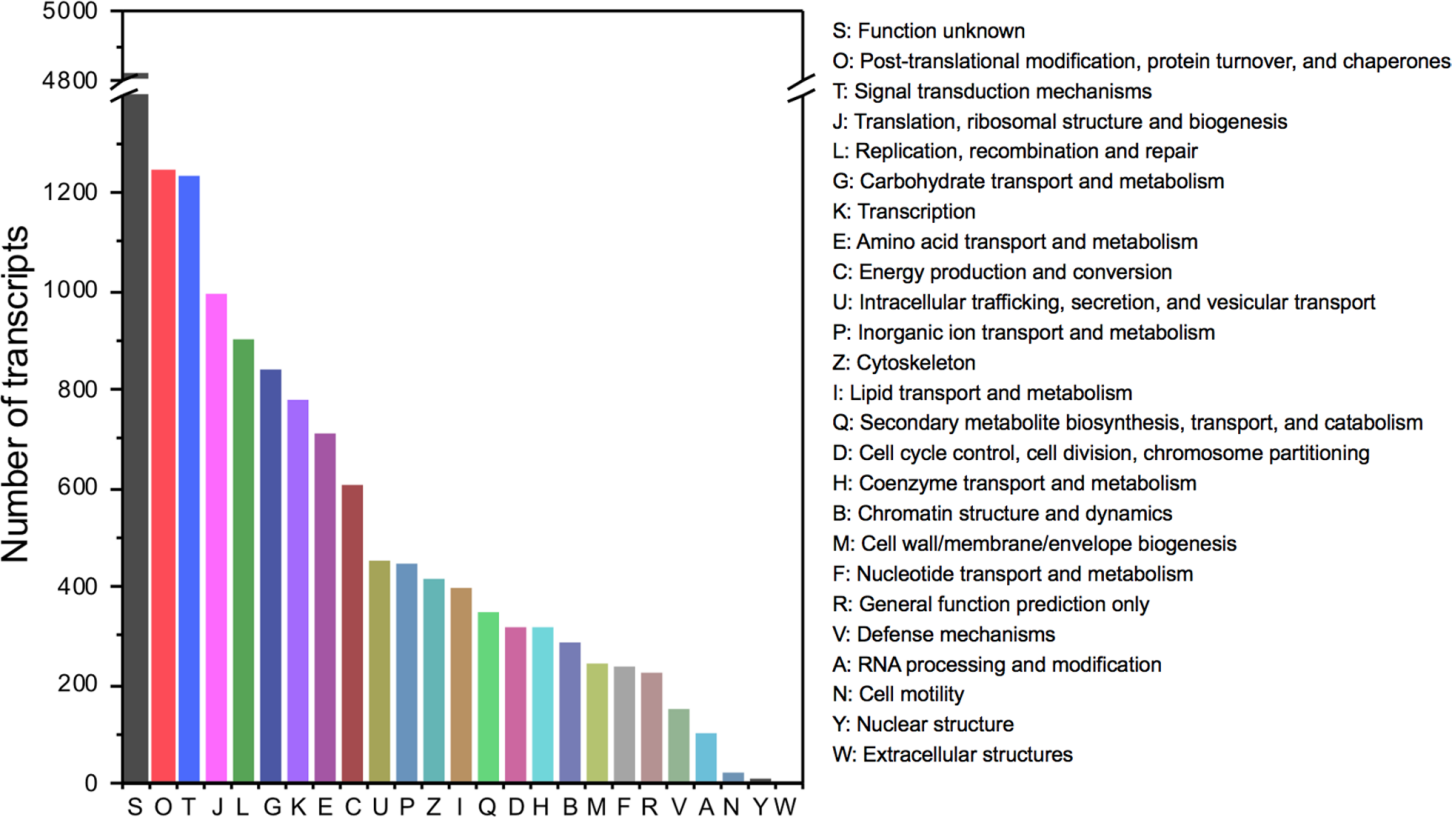
COG functional classification of transcripts of *S. mycoparasitica*. The functions of 15,346 transcripts were predicted and classified into 25 categories (indicated by the alphabetical notations) in descending order through the Clusters of Orthologous Groups (COG) classification

The functions of 15,346 transcripts were predicted and classified into 25 categories (resulting in the total number 16,054 since some of transcripts had assigned into more than one cluster) based on the Clusters of Orthologous Groups (COG) and EggNOG databases. As shown in Fig. 1, the largest cluster was ‘function unknown’ (4823, 30.04%), followed by ‘Post-translational modification, protein turnover, and chaperones’ (1246, 7.76%), ‘Signal transduction mechanisms’ (1230, 7.66%), ‘Translation, ribosomal structure and biogenesis’ (995, 6.20%), ‘Replication, recombination and repair’ (899, 5.60%), ‘Carbohydrate transport and metabolism’ (840, 5.23%), ‘Transcription’ (778, 4.85%), and ‘Amino acid transport and metabolism’ (706, 4.40%).

Other clusters, such as ‘Energy production and conversion’ (607, 3.78%), ‘Intracellular trafficking, secretion, and vesicular transport’ (447, 2.78%), ‘Inorganic ion transport and metabolism’ (446, 2.78%), ‘Cytoskeleton’ (413, 2.57%), ‘Lipid transport and metabolism’ (396, 2.47%), ‘Secondary metabolites biosynthesis, transport, and catabolism’ (344, 2.14%), ‘Cell cycle control, cell division, chromosome partitioning’ (317, 1.97%), ‘Coenzyme transport and metabolism’ (315, 1.96%), ‘Chromatin structure and dynamics’ (284, 1.77%), ‘Cell wall/membrane/envelope biogenesis’ (239, 1.49%), ‘Nucleotide transport and metabolism’ (232, 1.45%), and ‘General function prediction only’ (223, 1.39%) were less frequent.

Only a few transcripts (< 1% of the COG-annotated genes) were assigned to the clusters ‘Defense mechanisms’ (146, 0.91%), ‘RNA processing and modification’ (100, 0.62%), ‘Cell motility’ (20, 0.12%), ‘Nuclear structure’ (7, 0.04%), and ‘Extracellular structures’ (1, 0.01%).

### Gene Ontology (GO) functional classification

**Fig. 2:**
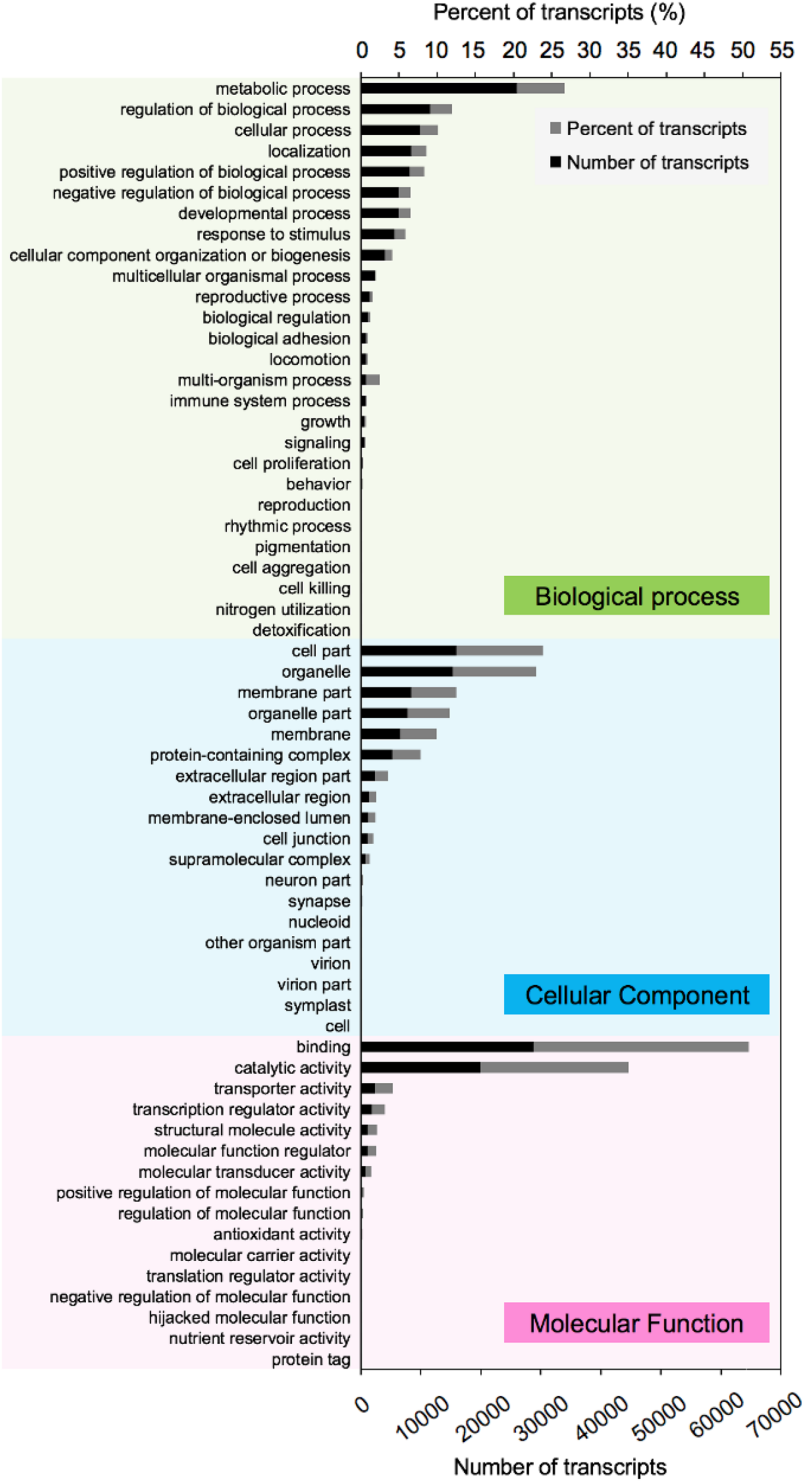
Gene Ontology (GO) functional classification or categorization of *S. mycoparasitica* transcripts. Each annotated sequence or transcript was assigned to terms within the three major categories: biological process, cellular component, and molecular function

The functions of the transcripts were predicted by a Gene Ontology (GO) analysis (http://geneontology.org/) in three functional categories: biological process, cellular component, and molecular function. A total of 25,668 transcripts (total number of GO: 220,543; many of the transcripts had more than one GO term; only 1393 transcripts had one GO term) were matched with 27 terms in the biological process group (97,265, 44.10%), 19 terms in the cellular components group (66,695, 30.24%), and 16 terms in the molecular function group (56,583, 25.66%), as shown in Fig. 2.

In the biological process (BP) category, ‘metabolic process’ (25,950, 26.68%) was the most dominant subcategory, followed by ‘regulation of biological process’ (11,594, 11.92%), ‘cellular process’ (9895, 10.17%), ‘localization’ (8389, 8.62%), ‘positive regulation of biological process’ (8122, 8.35%), ‘negative regulation of biological process’ (6336, 6.51%), and ‘developmental process’ (6319, 6.50%). The enriched subcategories were generally associated with the physiological and biological metabolism of the mycoparasite. For example, in the BP category, the terms ‘response to stimulus’ (5654, 5.81%), ‘cellular component organization or biogenesis’ (4059, 4.17%), ‘multicellular organismal process’ (2483, 2.55%), ‘reproductive process’ (1521, 1.56%), ‘biological regulation’ (1281, 1.32%), ‘biological adhesion’ (885, 0.91%), and ‘locomotion’ (877, 0.90%) may be related to the recognition, attachment, infection, and proliferation of the mycoparasite on the host. The fewest transcripts (13, 0.01%) were related to ‘detoxification’, which may be connected to the biodegradation of mycotoxins or secondary metabolites of *Fusarium* host.

In the cellular component (CC) category, the enriched terms included ‘cell part’ (15,905, 23.85%), ‘organelle’ (15,317, 22.97%), ‘membrane part’ (8354, 12.53%), ‘organelle part’ (7803, 11.70%), and membrane (6635, 9.95%). Unlike other parasitic fungi, *S.m.* is more closely related to the membrane part; however, the term ‘cell’ (2, 0.003%) was rarely observed.

In the molecular function (MF) category, the terms ‘binding’ (28,739, 50.79%) and ‘catalytic activity’ (19,867, 35.11%) were most highly enriched, followed by ‘transporter activity’ (2385, 4.22%). The subcategory ‘antioxidant activity’ (102, 0.18%) was related to few genes.

### Quantitative real-time PCR for validation of differentially expressed genes (DEGs)

**Fig. 3:**
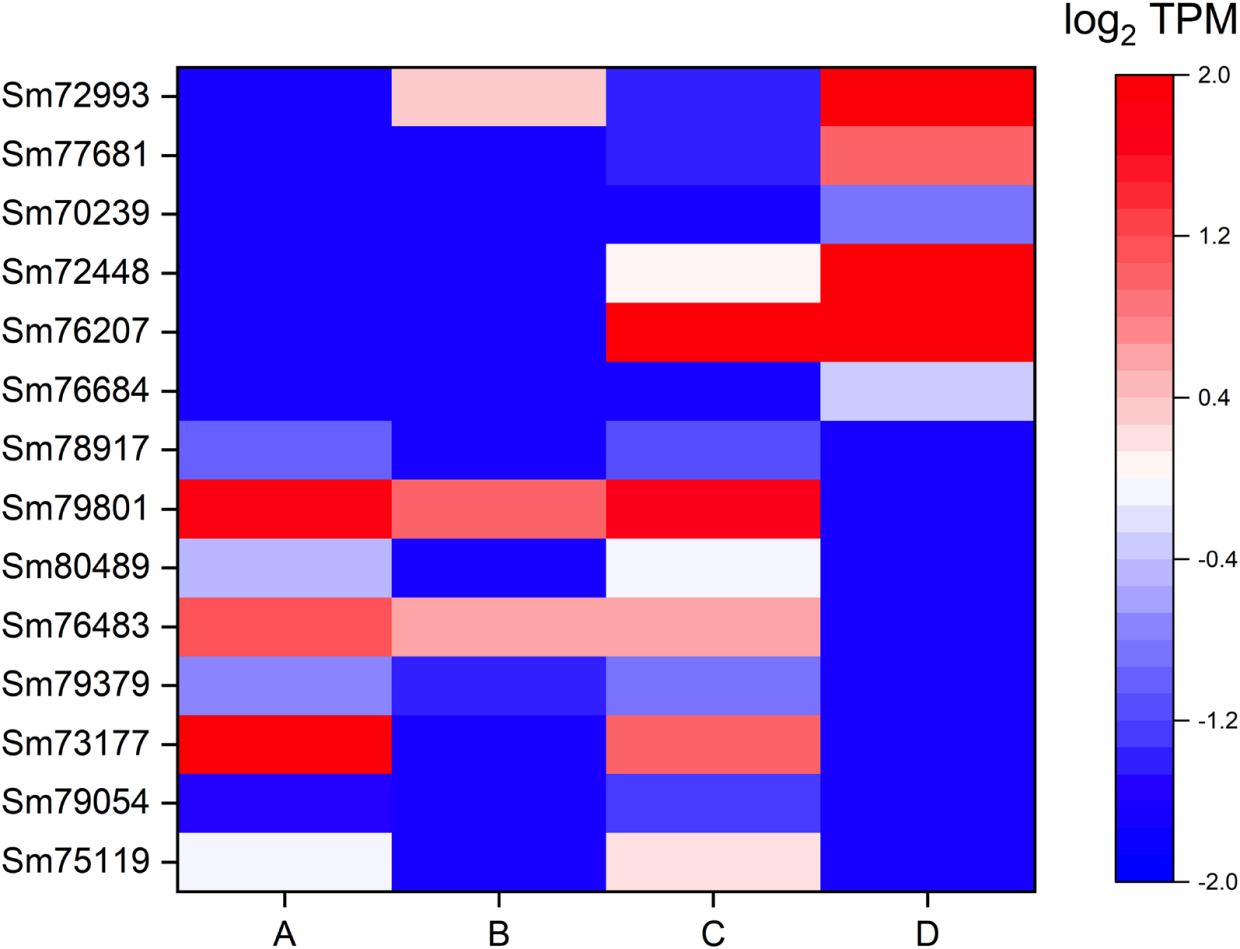
Heat map of 14 transcripts of *S. mycoparasitica* with/without the host *F. graminearum* at different incubation times (1.5 and 3.5 days). Colors from blue to red indicate that the transcript levels expressed as log_2_ TPM values from low to high. Each column represents each sample group (A, *S.m.* 1.5 days; B, *S.m.* 1.5 days interaction with *F.g.* 3-ADON; C, *S.m.* 3.5 days; D, *S.m.* 3.5 days interaction with *F.g.* 3-ADON)

**Fig. 4:**
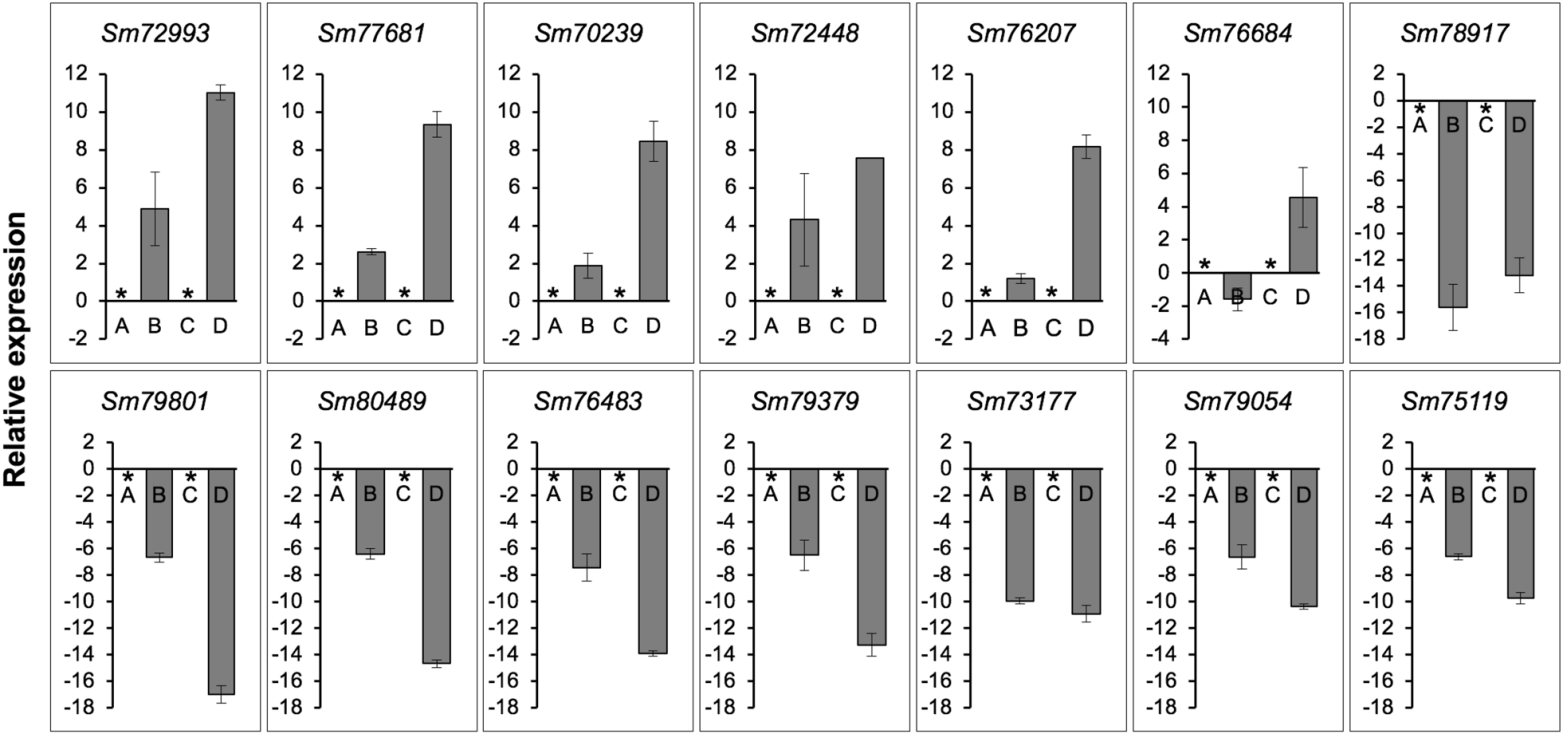
Relative expression levels of 14 transcripts of *S. mycoparasitica* determined by quantitative real-time PCR. with/without the host *F. graminearum* at different incubation times (1.5 and 3.5 days) determined by quantitative real-time PCR. A, *S.m.* 1.5 days; B, *S.m.* 1.5 days interaction with *F.g.* 3-ADON; C, *S.m.* 3.5 days; D, *S.m.* 3.5 days interaction with *F.g.* 3-ADON. A and C are compared with B and D, respectively. The *y*-axis represents the log_2_ fold change (A and C are zero indicated by the asterisk symbol). Error bars indicate the standard deviation of three replicates. The 18SrRNA was used as an internal control. Finally, the set of functional transcripts depicted Glucan 1,3-beta-glucosidase (*Sm72993*); Tfo1: Ac-like transposon (*Sm77681*); Glutathione synthetase (*Sm70239*); Endochitinase B (*Sm72448*); Filamentous hemagglutinin/adhesin (*Sm76207*); ATP-binding cassette (ABC) transporter (*Sm76684*); Uncharacterized mitochondrial protein ymf40 (*Sm78917*); RNase H domain-containing protein (*Sm79801*); Thioredoxin-2 (*Sm80489*); Glutathione *S*-transferase omega-like 2 (*Sm76483*); Thioredoxin reductase (*Sm79379*); NADPH–cytochrome P450 reductase (*Sm73177*); Uncharacterized protein (*Sm79054*); Uncharacterized membrane protein YJL163C (*Sm75119*)

Fourteen transcripts (*Sm72993*, *Sm77681*, *Sm70239*, *Sm72448*, *Sm76207*, *Sm76684*, *Sm78917*, *Sm79801*, *Sm80489*, *Sm76483*, *Sm79379*, *Sm73177*, *Sm79054*, and *Sm75119*) were selected based on expression levels (Fig. 3) and divided into two groups. In one group, six transcripts (*Sm72993*, *Sm77681*, *Sm70239*, *Sm72448*, *Sm76207*, and *Sm76684*) showed upregulated expression during the interaction with *F. graminearum* (or mycoparasitism) at 1.5 and/or 3.5 days. The transcripts encoded a glucan 1,3-beta-glucosidase (*Sm72993*), Tfo1: an Ac-like transposon (Tfo1) (*Sm77681*), glutathione synthetase (*Sm70239*), endochitinase B (*Sm72448*), filamentous hemagglutinin adhesin (FHA) (*Sm76207*), and ATP-binding cassette (ABC) transporter (*Sm76684*). The other group contained eight transcripts (*Sm78917*, *Sm79801*, *Sm80489*, *Sm76483*, *Sm79379*, *Sm73177*, *Sm79054*, and *Sm75119*) with decreased expression levels during the interaction with *F. graminearum* at both incubation times. They encode an uncharacterized mitochondrial protein ymf40 (*Sm78917*), RNase H domain-containing protein (*Sm79801*), thioredoxin-2 (*Sm80489*), glutathione *S*-transferase omega-like 2 (*Sm76483*) related to cell wall organization and biogenesis, thioredoxin reductase (*Sm79379*), NADPH-cytochrome P450 reductase (*Sm73177*) in ergosterol biosynthesis (biologically), uncharacterized protein (*Sm79054*), and uncharacterized membrane protein YJL163C, which may be related to the major facilitator superfamily (MFS) domain, and general substrate transporter (*Sm75119*). The qPCR results summarized in Fig. 4 were consistent with the RNA-sequencing results (TPM values), supporting the reliability of the sequencing results.

## Discussion

The rational classification of transcripts that encode proteins is critical for the effective utilization of genome sequences for functional and evolutionary studies (Tatusov et al. 2000). This is of particular importance for microbial genome analyses (Galperin et al. 2019) aimed at improving fungal and bacterial biotechnology as well as applications in agricultural settings to produce food commodities (Ganeshan et al. 2021).

Despite being widespread, mycoparasitism is probably not ancestral within the kingdom Fungi. Nevertheless, the necrotrophic generalist paradigm is based on soil-born fungi (Domsch et al. 1980; Schroers et al. 1999), such as *Clonostachys* and *Trichoderma* (Sun et al. 2015), as evolutionary distinct and more ancient fungal hyperparasites compared with biotrophic *Sphaerodes/Melanospora* hyperparasites and specialists. Accordingly, *S. mycoparasitica* is a useful reference point from which to discuss transcriptomic divergence across fungal taxa with variation in mycoparasitic biocontrol behavior, cellular organization, and ecological lifestyles.

Generally, a series of cellular activities and genetic regulatory mechanisms are involved in the mycoparasitic process. These include recognition–attachment and penetration–parasitism, and these events in *S. mycoparasitica* are triggered at different stages of parasitism on *Fusarium* hosts (Vujanovic and Goh 2010). Here, we present the first functional transcript profile of the biotrophic mycoparasitism of *S.m.* on *F. graminearum* 3-ADON host. While the functional classification of transcripts displays *S. mycoparasitica* polyphagous features among distinctive mycoparasitic lifestyles, the ontology and differential expression of genes remark series of processes related to biocontrol, resistance, and detoxification of mycotoxins. Fusarium production of several types of toxins dispersed throughout food chain provides to the *S.m.*-BCA multiple advantages in positive modulation of the health risk for humans and animals.

### Functional classification (Clusters of Orthologous Groups)

In this study, in the interactive RNA-Seq *S.m.* profile, the largest number of transcripts were assigned to the group ‘function unknown’, suggesting that a substantial portion of the basic molecular mechanisms underlying biotrophic mycoparasitism have yet to be discovered (Naranjo-Ortiz and Gabaldón 2019). The ‘general function prediction only’ is the largest functional group reported in the genomes of various necrotrophic mycoparasites [*T. atroviride* (1246 of 7648, 16.29%); *T. virens* (1307 of 7875, 16.60%); *T. reesei* (1030 of 6833, 15.07%); *T. brevicompactum* (1937 of 12058, 16.06%); *C. rosea* (3489 of 8615, 40.5%)], with ‘function unknown’ accounting for fewer transcripts [*T. atroviride* (397 of 7648, 5.19%); *T. virens* (400 of 7875, 5.08%); *T. reesei* (383 of 6833, 5.61%); *T. brevicompactum* (587 of 12,058, 4.87%); *C. rosea* (1602 of 8615, 18.60%)] (Shentu et al. 2014; Sun et al. 2015). An improved knowledge of the relationship between mycoparasitic lifestyles and biocontrol behaviors seems vital for the selection of bioinoculants for the control of *F.* *graminearum,* a cause of Fusarium head blight (Kim and Vujanovic 2016). Further, the clusters ‘Post-translational modification, protein turnover, and chaperones’ (7.76%) and ‘Signal transduction mechanisms’ (7.66%) were the second and third largest groups, similar to results for the three *Trichoderma* species [*T. atroviride* (8.11% and 6.88%); *T. virens* (7.94% and 7.02%); *T. reesei* (8.18% and 7.55%)]. However, for *C. rosea* and *T. brevicompactum,* the second and third largest groups were the clusters for ‘transcription’ and ‘carbohydrate transport and metabolism’ [*C. rosea* (24.20% and 22.10%); *T. brevicompactum* (7.94% and 7.44%)]. Additionally, the smallest group was the cluster ‘Extracellular structures’ *S.m.,* while the clusters ‘nuclear structure’ in *C. rosea* and *T*. *brevicompactum* and “cell motility” in other three *Trichoderma* species were the least well-represented.

The similarities and differences in COG classification between *S. mycoparasitica* and other fungi seem to be coordinated with their lifestyles (i.e., mycoparasitic *T. atroviride*, *T. virens,* and *C. rosea* and saprophytic *T. reesei* and *T. brevicompactum*). However, the polyphagous behavior of *S.m.* against fungal species within the genus *Fusarium* contributed to additional differences in its transcriptomic profile compared with those of mycoparasitic generalists. Further comparative proteomics and secretomic analyses are needed to better explain these relationships.

### Functional (Gene Ontology) classification

Enzymatic processes using biocatalysts play an important role in the biocontrol efficiency of mycoparasites. The enrichment for ‘binding’ and ‘catalytic activity’ may be crucial for the mode of action during mycoparasitism. Enrichment for ‘binding’, ‘catalytic activity’, and ‘transporter activity’ has also been reported in *Clonostachys rosea* 67–1 against *Sclerotinia sclerotiorum* (Sun et al. 2015). The overrepresentation of ‘transporter activity’ (including ABC transporters) has also been found in zearalenone-induced *Clonostachys rosea* (Kosawang et al. 2014a), which might be connected to our research on the biodegradation of zearalenone by *S. mycoparasitica* (Kim and Vujanovic 2017).

### Quantitative real-time PCR and differentially expressed genes

Several mycoparasitism-related differentially expressed genes (DEGs) have been reported. Signal transduction or signaling-related proteins, including G-proteins and mitogen-activated protein kinases (MAPKs), are involved in a series of processes, e.g., recognition or sensing the host or prey, (interspecific) communication, and attachment of parasitic appressorium-like structure(s) to the host (Omero et al. 1999; Reithner et al. 2007; Sun et al. 2020; Yang 2017; Zeilinger et al. 2005). In the *S. mycoparasitica* transcriptome, the expression levels of transcripts encoding G-proteins and MAPKs were higher during the interaction with the host than in controls (without host interactions) (data are not shown).

During the interaction between *S. mycoparasitica* and *Fusarium* host, the expression of the transcript (*Sm76207*) annotated as a filamentous hemagglutinin adhesin increased. We assumed that the adhesin may facilitate the attachment process by self-cell–cell adhesion and/or host cell-surface adherence and contribute to the virulence and infectivity (Bernardi et al. 2018).

Cell wall-degrading enzymes (e.g., endochitinases, endoglucanases, and serine proteases) are required not only for penetration for successful mycoparasitism but also for cell wall remodeling during active growth, recycling during aging, and autolysis of cell wall components (Carsolio et al. 1994; Geremia et al. 1993; Gruber and Seidl-Seiboth 2012; Reithner et al. 2011; Thrane et al. 1997). *S. mycoparasitica* showed the upregulation of *Sm72448* and *Sm72993* encoding endochitinase B and glucan 1,3-beta-glucosidase, respectively. In the *S.m.* transcriptome, 31 transcripts were annotated to 19 chitinases (glycoside hydrolase family 18) or chitinase-related proteins (*ChiA*, *ChiA1*, *ChiB*, *ChiB1*, etc.). However, the number of genes is not likely to be correlated with the catalytic efficiency of enzymes on certain substrates. In a broad comparison, the number of chitinase-encoding genes of *S.m.* is intermediate between those of the mycoparasitic *C. rosea* (14) and *Trichoderma* species (29 in mycoparasitic *T. atroviride,* 36 in mycoparasitic *T. virens*, and 20 in saprophytic *T. reesei*), reviewed by Gruber and Seidl-Seiboth (2012). In particular, *chiB1* of *C. rosea* is induced during fungal–fungal interactions and is highly induced by colloidal chitin (Tzelepis et al. 2015). The presence of chitinases in *S.m.* further implies that hydrophobic cell wall proteins (e.g., hydrophobins) and carbohydrate-binding proteins are involved in the protection of its own cell wall to manage the accessibility of chitins during hyphal development.

Toxin-related transcripts were also upregulated during the mycoparasitism, including toxin-encoding genes (e.g., killer toxin subunits alpha/beta) and toxin transport-encoding genes (e.g., putative HC-toxin efflux carrier TOXA), which may facilitate the mycoparasitism. These killer toxin-like chitinases have been reported in *Trichoderma* and *Clonostachys*.

Mycoparasites might express genes to effectively regulate biological functions related to the host interaction, such as genes related to the response to stimulus (stress response), secondary metabolite metabolisms, and detoxification (defense mechanism). In this regard, the ABC transporter is an important membrane protein for the uptake and efflux of various molecules. In the case of another mycoparasite, *C. rosea* IK726, ABC transporter G5 is involved in the detoxification of *Fusarium* mycotoxins (e.g., zearalenone) and xenobiotics (e.g., fungicides), resulting in overall cell protection (Dubey et al. 2014; Kosawang et al. 2014b; Víglaš and Olejníková 2021). Regarding the *S. mycoparasitica* interactive transcriptome, the transcripts for different types of ABC transporters, including the ABC G superfamily, were expressed and might be involved in the detoxification or transformation of *Fusarium* mycotoxins. Recently, it was reported that ABC transporters along with MFS may play important roles during the mycoparasitism of *Coniothyrium minitans* on *Sclerotinia sclerotiorum* (Zhao et al. 2020). According to Nygren et al. (2018), during interactions between *C. rosea* and both *Botrytis cinerea* and *F. graminearum,* the gene encoding hydantoinase/oxoprolinase, which is involved in glutathione biosynthesis, was upregulated. The expression of *abcG18* in *C. rosea* was significantly increased during the interaction with *F. graminearum*, implying a specific response to *F. graminearum*. Many of the genes encoding MFS transporters were also highly expressed during the interaction with *F. graminearum*; however, this was not a focus of the study.

In addition to ABC transporters, our results revealed various functional groups of genes, such as glutathione *S*-transferases (along with glutathione synthetase and glutathione reductase) as well as cytochrome P450 enzymes (with NADPH-cytochrome P450 reductase), with potential roles in detoxification and stress response in the active defense system (Gullner et al 2018). In the transcriptome of *S. mycoparasitica*, the expression levels of these transcripts were altered during the interaction with the *Fusarium* host. Further, the diverse thioredoxin (proteins)-encoding genes showed variation in gene expression levels. According to Fernandez and Wilson (2014), the glutathione and thioredoxin antioxidation systems in *Magnaporthe oryzae* could facilitate biotrophic colonization and are potential determinants of rice blast disease. Glutathione reductase is required for neutralizing plant-generated reactive oxygen species (ROS) and thioredoxin proteins contribute to cell-wall integrity (Fernandez and Wilson 2014). In *S. mycoparasitica*, the thioredoxin and thioredoxin reductase may differ from those of *M. oryzae* or from those affected by glucose availability, since they were downregulated in the *S.m.* transcriptome.

In the future, detailed comparative analyses could be applied to various sets of specific genes related to mycoparasitism to elucidate the distinct molecular mechanisms underlying biotrophic mycoparasitism in *S. mycoparasitica* for its polyphagous activity against *Fusarium* species related to Fusarium head blight (FHB). In order to understand this specific mycoparasitism–FHB system, mobile elements or transposons such as the activator (Ac) Tfo1 discovered in *S. mycoparasitica* that also occurs in its fungal (*Fusarium*) and plant (Gramineae) hosts (Okuda 1998) deserve further study through the food chain.

## Conclusions

In this study, we conducted transcriptomic analyses of the early hyphal–hyphal interaction of *Sphaerodes mycoparasitica* to get to know the biotrophic mycoparasitism against *Fusarium graminearum*. De novo assembly from RNA-Seq data and functional annotation of transcriptome were performed, while qPCR was used to confirm the reliability of the sequencing results. The RNA-Seq profile demonstrates that the specific-biotrophic mycoparasitism is a complex process. Indeed, a broad range of transcripts with known (e.g., endochitinases, glucanases, serine proteases, adhesins) and unknown functions are displayed by the mycoparasite to successfully invade *F. graminearum*. We discovered a plethora of ABC transporters which suggests that S*. mycoparasitica* has particular mycoparasitism-associated ability to sustain toxic environment or detoxify mycotoxins made by *F. graminearum*. Interestingly, *S. mycoparasitica* downregulated expression of thioredoxin reductase gene required for antioxidant stress resistance which is an opposite finding about this gene compared to mycoparasitic generalists. These findings indicate that *S. mycoparasitica* has remarkable resistance in combatting *Fusarium*, which was not previously demonstrated or reported elsewhere. Taken together, our study results provide novel insights into possible mechanisms underlying antioxidant roles of ABC, glutathione synthetase and cytochrome P450 genes combined with pathogenicity related degradative enzymes in *S. mycoparasitica* for controlling *F. graminearum*. This RNA-Seq study results enhance opportunity to future discoveries in harnessing the full potential of this specific-mycoparasitism, its evolutionary features, and biocontrol advantages.

## Data Availability

All relevant data and material are presented in this paper. The datasets generated during and/or analyzed during the current study are available from the corresponding author on reasonable request.

## Abbreviations

NGS: Next-generation sequencing
qPCR: Quantitative real-time PCR
RNA-Seq: RNA sequencing
DEGs: Differentially expressed genes
COG: Clusters of Orthologous Groups
GO: Gene Ontology
ROS: Reactive oxygen species
